# Turtles all the way down: do biological mechanisms for epidemiological observations always matter?

**DOI:** 10.1007/s10654-021-00807-8

**Published:** 2021-10-07

**Authors:** David T. P. Buis, Jos van Roosmalen

**Affiliations:** 1 Department of Internal Medicine, Amsterdam Infection and Immunity Institute, Amsterdam UMC, Vrije Universiteit Amsterdam, De Boelelaan 1117, 1081 HV Amsterdam, The Netherlands; 2 Athena Institute, VU University, Amsterdam, The Netherlands; 3 Department of Obstetrics and Gynaecology, Leiden University Medical Center, Leiden, The Netherlands

**Keywords:** Epidemiology, Causality, Causal inference, Hill's criteria, Biological mechanisms

## Hill’s plausibility criterion

Biological mechanisms are ubiquitous in reports of epidemiological research. Many researchers extensively elaborate on a biological potential mechanism underlying their findings under the assumption that the presence of one is somehow related to the quality of their work. This interesting phenomenon echoes the work of Austin Bradford Hill, the famous epidemiologist who coined the Hill’s criteria for judgement of causality in epidemiological research. In Hill’s own words they are criteria from all of which “we should study association before we cry causation” [1]. They include strength, consistency, specificity, temporality, biological gradient, plausibility, coherence, experiment and analogy. All Hill’s criteria are almost never systematically used to judge whether associations represent causal effects. Hill’s plausibility criterion, however, is still frequently used to strengthen or weaken causal claims in epidemiological studies. Höfler phrased this criterion as “the observed association can be plausibly explained by substantive matter (e.g. biological) explanations” [2]. The exact definition of the term “mechanism” is topic of ongoing philosophical debate. In this viewpoint we define a mechanism of a behavior as “a complex system which produces that behavior by the interaction of a number of parts” as proposed by Glennan [3]. For the sake of the argument, we make a somewhat arbitrary distinction between “biological mechanisms” taking place within individual organisms, in contrast with “social” mechanisms that occur to populations [4]. While reading epidemiological reports, one cannot help feeling that epidemiologists highly value these biological mechanisms. Why is that and is it always justified?

## Open and closed systems

The strong urge of epidemiologists to back up their causal claims by presenting biological mechanisms could be explained by the limitations that are posed by empirical research in relatively open systems. Scottish philosopher David Hume posed the idea of open systems which always require external information making definite proof impossible [5]. On the contrary, systems such as mathematics are considered “closed” since they require only a few axioms to start from. One of the key features of a closed system is its deterministic behavior, i.e. all future states are fully determined by the current condition of the system and as a consequence there is only one possible course of events [6]. In comparison with basic laboratory research, epidemiological studies are generally more “open” since the conditions are less controllable. Imagine for example a randomized controlled trial (RCT) investigating length of antibiotic treatment for a certain type of infection. Patients are allocated in a 1:1 fashion to 28 days (intervention group) or 42 days (control group) of antibiotic treatment with 3-months all-cause mortality as outcome. Assuming this RCT is perfectly executed, i.e. blinded, without loss to follow-up and with full compliance to the intervention, it is possible to estimate a population effect. However, the open character of the study makes it impossible to predict at baseline the outcome of an individual study participant X. It is a realistic possibility that this patient X dies of causes totally unrelated to the study, e.g. a car accident, after 2 months. This event would be impossible to predict at baseline, even while one assumes knowledge of all relevant medical factors [6]. Laboratory studies, on the other hand, are generally more closed since researchers are able to control most conditions relevant for the experiment. Therefore, causal inference in epidemiology represents a greater challenge and a potential biological mechanism explaining the observations could provide necessary backup to support an otherwise weak causal claim.

## Bayesian reasoning

Furthermore, many epidemiologists include biological mechanisms in “Bayesian reasoning” to weigh their findings in relation to previous research. Using Bayes’ theorem a known or plausible biological mechanism increases the probability that an association estimated in an epidemiological study represents a true effect [7]. For the purpose of causal inference, however, a very clear distinction should be made between the ability of an individual study to support a causal claim and weighing the body of available evidence to do so. For the latter the results of all studies concerning the research topic, whether epidemiological or basic laboratory research, are relevant. Moreover, judging the presence of confounding and bias in epidemiological studies is frequently based on reasoning that involves mechanisms. The quality of the epidemiological study itself, however, is fully independent of the results of other studies [8]. Therefore, epidemiologists should be very careful to support an otherwise weak causal claim from an epidemiological study with potential biological mechanisms inferred from other studies. As Savitz phrases it: “Does a poor epidemiologic study improve over time if the evidence from laboratory research becomes stronger?” [8]. Moreover, the plausibility criterion can by definition only support epidemiological findings that have a known or plausible biological mechanism. Therefore using this criterion to judge whether a causal association exists is at risk for conservatism towards new associations that have no foundation (yet) in fundamental research, while one could argue that in these situations the added value of epidemiology to our understanding of the world is the greatest.

Moreover, there is no standard framework to guide incorporation of biological mechanisms in assessment of strength of evidence for causality. Frequently used approaches as meta-analyses, which combine results from similar study designs, and triangulation, which aims to integrate data from different methodological approaches, mainly focus on weighing evidence from multiple epidemiological studies [9]. These frameworks do not offer much guidance on how to combine results from epidemiological studies with potential underlying biological mechanisms. As a result, researchers seem free to propose a biological mechanism that fits with the results from their epidemiological study, which is at risk of cherry picking and biased causal claims.

## Counterfactual reasoning

Hill’s criteria are not the only paradigm used for judging whether or not phenomena are causally related. It is safe to say that in current epidemiological practice the counterfactual framework forms the dominant paradigm in judgement of causality, especially in studies of interventions. The counterfactual framework states that A is a cause of B when B differs between the situation that A is present or not [10]. This idea is consistent with human reasoning in matters of cause and effect: comparing the outcome between A present and A absent. In the ideal experiment, i.e. randomized, blinded, without loss to follow-up and with full compliance to the intervention, a group of participants with A present and a group with A absent can be considered exchangeable. As a consequence, in this situation association equals causation and it is possible to estimate a population causal effect. All epidemiological and statistical approaches to prevent or adjust for confounding and selection bias can be understood from the counterfactual framework as disruptive factors for obtaining exchangeability between groups [10]. In the context of biological mechanisms it should be noted that counterfactual reasoning does not require such a mechanism for determination of causality. Assuming no random error, estimation of a difference between A present and A absent is sufficient for causal inference in the counterfactual framework, assuming exchangeability between both situations. Therefore, the ability of an individual epidemiological study to assess causality should be judged in terms of factors influencing the degree of exchangeability between groups, i.e. confounding and bias, and is in no way related to biological mechanisms inferred from other studies.

## The sum of the parts

Despite the emphasis on biological mechanisms in epidemiological reports, in many instances we do not know the underlying mechanism of an association. For example, anosmia is a frequent symptom in COVID-19 patients of which the pathophysiology is still unclear [11]. Few people, however, would doubt that COVID-19 is a cause of this phenomenon. This is the reason why Hill attached relatively little value to plausibility as explanation. In his original paper is written that “it will be helpful if the causation we suspect is biologically plausible. But this is a feature I am convinced we cannot demand. What is biologically plausible depends upon the biological knowledge of the day” [1]. This statement emphasizes that our knowledge about the natural world is limited and lack of a known underlying biological mechanism today does not preclude causality. This is especially true in studies of complex interventions in heterogeneous populations, in which the mechanism is a complicated interaction between multiple entwined biological, behavioral and social factors [12]. One could argue that this is not problematic for the process of causal inference: if there is a biological mechanism causality is more probable than when there is none. This argument relies on the assumption that all phenomena can be fully reduced to events happening on a small scale, e.g. interactions between molecules. Many scientists apply a reductionist approach in their research, but to what degree science is a truly reductionist endeavor is open for debate [13]. There are many examples where epidemiological observations could not be reduced to biological mechanisms. These mechanisms do not explain complex phenomena such as risky behavior of HIV-positive individuals or the impact of neighborhood conditions on cardiovascular risk [14, 15]. In these cases reducing the observations to events happening within individuals would not lead to a deeper understanding of reality. In this context Ernst Mayr is frequently quoted who wrote that “when two entities are combined at a higher level of integration, not all the properties of the new entity are necessarily a logical or predictable consequence of the properties of the components" [16]. Sometimes in the sum of the parts, there is more than the parts.

## Mechanisms at multiple levels

From an epistemological perspective the distinction between epidemiological observations and their biological explanations is in some aspects arbitrary, since the mechanism itself also requires an underlying mechanism. Consider for example a study examining the effect of lowering systolic blood pressure on stroke [17]. We could imagine the following simplified potential biological mechanism: decreasing the pressure in vessels reduces the risk that these vessels tear and result in hemorrhagic stroke. This explanation seems plausible, but would immediately raise the question how this exactly works on a cellular or even molecular level. This line of reasoning would result in a classic *turtles all the way down* situation in which every proof requires another underlying proof making definite proof impossible because of infinite regression. Searching for an underlying mechanism at a continuously lower level would inevitably run to an end when one encounters fundamental laws of physics, e.g. the law of universal gravitation, that cannot be explained. This is not problematic for the practical implications of the study, since knowing the molecular mechanism is not necessary to design an effective intervention to prevent stroke. It could, however, be problematic for the purpose of causal inference if one adheres to the notion that a definite mechanism is necessary to prove causality.

## Practical epidemiology

Lastly, a distinction exists between the scientific and more practical purpose of epidemiology. The first goal could be defined as to find true causal effects using epidemiological observations and the latter as using these observations to inform health policies to reduce morbidity and mortality. Sometimes knowing the underlying biological mechanisms helps to design interventions, e.g. chemotherapy directed at specific mutations in tumor cells [18]. In many instances, however, knowing biological mechanisms is by no means indispensable in using epidemiological observations to inform health policies. This is illustrated by the successful efforts of John Snow, one of the founders of epidemiology, to fight cholera in nineteenth century London. By laborious investigations Snow was able to establish an association between the water pump on Broad Street and the spread of cholera [19]. Subsequent removal of the force rod was successful in restricting spread of the epidemic. Snow was able to reach this major achievement of improving public health while being totally unaware of germ theory, which was only developed later.

A similar example in that time occurred in Wenen, where physician Louis-Ferdinand Semmelweis detected the importance of hand-washing during childbirth to prevent puerperal sepsis [20]. In his maternity clinic, births were guided by medical students and in another clinic by midwives. Semmelweis observed that incidence of death from maternal sepsis was much higher in the ward run by students, who physically examined pregnant women after they had practiced autopsy. When medical students were posted in the other maternity, death from sepsis moved out with them to that clinic. Semmelweis succeeded to find proof in a before and after study that disinfection by handwashing during birth greatly reduced incidence of maternal sepsis. Obstetric elites in Western Europe could not accept Semmelweis’ findings, however, which eventually led to his discharge from work.

Both Snow and Semmelweis did not know the biological mechanism of their observations. Snow’s proposed strategy was, however, immediately implemented by the local authorities while it took another fifty years before most leading obstetricians were convinced of the benefits of disinfection by handwashing at the expense of many deaths. Their stories beautifully illustrate that knowing the exact biological mechanism is not always a necessity to be a successful epidemiologist.

## References

[1] Hill AB (1965). The environment and disease: association or causation?. Proc R Soc Med.

[2] Höfler M (2005). The Bradford Hill considerations on causality: a counterfactual perspective. Emerg Themes Epidemiol.

[3] Glennan SS (1996). Mechanisms and the nature of causation. Erkenntnis.

[4] Weed DL, Hursting SD (1998). Biologic plausibility in causal inference: current method and practice. Am J Epidemiol.

[5] Rosendaal FR (2016). The p-value: a clinician's disease?. Eur J Internal Med.

[6] Dekkers OM, Mulder JM (2020). When will individuals meet their personalized probabilities? A philosophical note on risk prediction. Eur J Epidemiol.

[7] Rembold CM, Watson D (1988). Posttest probability calculation by weights. A simple form of Bayes' theorem. Ann Internal Med.

[8] Savitz DA (1994). In defense of black box epidemiology. Epidemiology.

[9] Lawlor DA, Tilling K, Davey SG (2017). Triangulation in aetiological epidemiology. Int J Epidemiol.

[10] Hernán MA (2004). A definition of causal effect for epidemiological research. J Epidemiol Community Health.

[11] Han AY, Mukdad L, Long JL, Lopez IA (2020). Anosmia in COVID-19: mechanisms and significance. Chem Senses.

[12] Kotaska A (2004). Inappropriate use of randomised trials to evaluate complex phenomena: case study of vaginal breech delivery. BMJ.

[13] Dupré J. It is not possible to reduce biological explanations to explanations in chemistry and/or physics. In: Contemporary debates in philosophy of biology; 2009. p. 32–47.

[14] Diamond C, Buskin S (2000). Continued risky behavior in HIV-infected youth. Am J Public Health.

[15] Barber S, Hickson DA, Wang X, Sims M, Nelson C, Diez-Roux AV (2016). Neighborhood disadvantage, poor social conditions, and cardiovascular disease incidence among African American adults in the Jackson heart study. Am J Public Health.

[16] Mayr E. Cause and effect in biology: kinds of causes, predictability, and teleology are viewed by a practicing biologist. 1961;134(3489):1501–6. 10.1126/science.134.3489.1501 14471768

[17] Rojas-Saunero LP, Hilal S, Murray EJ, Logan RW, Ikram MA, Swanson SA (2021). Hypothetical blood-pressure-lowering interventions and risk of stroke and dementia. Eur J Epidemiol.

[18] Green MR (2004). Targeting targeted therapy. N Engl J Med.

[19] Fine P, Victora CG, Rothman KJ, Moore PS, Chang Y, Curtis V (2013). John Snow's legacy: epidemiology without borders. Lancet.

[20] Céline L-F. Het leven en werk van Philipp Ignaz Semmelweis (1818–1865). Academic Thesis, Paris, translated from French, translated by Jan Versteeg. Uitgeverij De Arbeiderspers, Amsterdam 1986.

